# Association of serum levels of FGF23 and α-Klotho with glomerular filtration rate and proteinuria among cardiac patients

**DOI:** 10.1186/1471-2369-15-147

**Published:** 2014-09-08

**Authors:** Michishige Ozeki, Shu-ichi Fujita, Shun Kizawa, Hideaki Morita, Koichi Sohmiya, Masaaki Hoshiga, Nobukazu Ishizaka

**Affiliations:** Department of Cardiology, Osaka Medical College, Takatsuki-shi Daigaku-machi 2–7, Osaka, 569-8686 Japan

**Keywords:** Fibroblast growth factor-23, Klotho, Chronic kidney disease

## Abstract

**Background:**

Expression and/or excretion of fibroblast growth factor-23 (FGF23) and its co-receptor Klotho are altered in patients with end-stage renal disease. The possibility that the FGF23/α-Klotho system mediates the aggravated cardiovascular outcome among patients with chronic kidney disease (CKD) has been suggested. We determined whether FGF23 and α-Klotho concentrations are altered among patients with reduced renal function and proteinuria.

**Methods:**

Serum FGF23 and α-Klotho were measured in cardiology patients who were not undergoing chronic hemodialysis. Estimated glomerular filtration rate (eGFR) was correlated negatively with FGF23 and positively with α-Klotho.

**Results:**

The correlation between FGF23 and the renal tubular maximum reabsorption rate of phosphate to the GFR (TmP/GFR) was not significant, but that between FGF23 and serum calcium or inorganic phosphate was significant among patients with an estimated GFR of less than 60 mL/min/m^2^. By stepwise multivariate regression analysis, eGFR was selected as significant predictor for FGF23 or α-Klotho among patients with an estimated GFR of less than 60 mL/min/m^2^; however, urine albumin/creatinine ratio was not selected as a predictor for FGF23 or α-Klotho irrespective of the eGFR levels. In patients with eGFR of <60 mL/min/1.73 m^2^, UACR was significantly associated with log(FGF23); but, this association did not remain statistically significant in a multivariate model.

**Conclusions:**

Among cardiology patients with various stages of CKD, serum concentrations of FGF23 and α-Klotho were associated with renal function, but not with the extent of proteinuria.

## Background

Fibroblast growth factor-23 (FGF23) plays a crucial role in the regulation of calcium-phosphate metabolism by suppressing the renal tubular reabsorption of phosphate via activation of FGFR-1c in the presence of its co-receptor Klotho, which was originally identified as an anti-aging molecule [1–3]. Among patients with end-stage renal disease, serum levels of FGF23 increase in response to elevated serum phosphorus, and those of α-Klotho decrease. Among patients with end-stage renal disease, serum levels of FGF23 increase in response to elevated serum phosphorus. In addition, serum α-Klotho decreases with the progression of renal dysfunction [4], which may be attributed to the fact that the membrane protein klotho is expressed predominantly in kidney and brain and that renal expression of klotho is decreased in patients with chronic kidney disease (CKD) [5, 6]. FGF23 is presumed to exert extrarenal manifestations [7], including left ventricular hypertrophy and cardiac systolic dysfunction [8, 9]. Together with the observation that reduced α-Klotho is associated with coronary artery disease [10], these findings suggested that modulation of FGF23/α-Klotho may represent one of the crucial factors underlying the cardiac [11] and vascular remodeling observed in patients with CKD.

In our previous analyses, we found that FGF23 is associated with left ventricular hypertrophy and cardiac systolic dysfunction [12, 13]. As a result, therapies that, directly or indirectly, lower serum levels of FGF23 and, in reverse, elevate the serum α-Klotho level, might represent a novel target to slow the cardiac remodeling process [14]. Before searching for effective therapeutic strategies, however, we should analyze what determines FGF23/α-Klotho levels in cardiology patients; only a little information is available about FGF23/α-Klotho in non-CKD patients [15] as compared with patients in cardiology patients undergoing hemodialysis [16, 17].

CKD is defined to be present when low glomerular filtration rate (GFR) and/or enhanced urinary protein excretion, both of which conditions has been shown to be associated with cardiac remodeling [18, 19], exists for certain period of time. However, relationship between FGF23/α-Klotho and proteinuria seems to have been less extensively examined thus far, as compared with that between GFR and FGF23/α-Klotho [20]. To this end, in the current study, we investigated the association of the extent of proteinuria, as well as eGFR, with circulating levels of α-Klotho and FGF23 among cardiology patients.

## Methods

### Study population

The current retrospective study was approved by the Ethics Committee of Osaka Medical College. Between October 2012 and January 2014, 190 cardiac inpatients were recruited who provided written informed consent and for whom sufficient information regarding the data analysis was available. After excluding five patients undergoing chronic hemodialysis, 185 patients were enrolled in the current study.

### Laboratory analysis

Aliquots of serum and plasma were obtained and stored immediately at −80 degrees until use. Calcium (Ca), inorganic phosphate, C-reactive protein (CRP), and B-type natriuretic peptide (BNP) were measured by routine laboratory methods. When serum albumin was 4 mg/dL or lower, serum Ca levels were corrected by the formula: Ca + (4–[serum albumin]), and reported as corrected Ca (cCa). Serum levels of intact FGF23 were measured using a two-step FGF23 enzyme-linked immunosorbent assay (ELISA) kit (Kainos Laboratories Inc., Tokyo, Japan), and serum levels of soluble α-Klotho were measured using a solid-phase sandwich ELISA kit (Immuno-Biological Laboratories, Gunma, Japan) according to the manufacturer’s instructions. The interclass correlation coefficients (ICC) of intra- and inter-operator reliability for α-Klotho were 0.97 and 0.94, respectively, those for FGF23 have been described elsewhere [13]. The eGFR was calculated by the following Modification of Diet in Renal Disease equation for Japanese subjects: eGFR = 194 × (serum creatinine)^-1.094^ × (age)^-0.287^ [21]. Urinary albumin in spot urine was measured by turbidimetric immunoassay and corrected to the urinary albumin level, and expressed as mg of albumin per 1 g of creatinine (urine albumin creatinine ratio, UACR).

Fractional excretion of calcium (FEca) was calculated by the formula: FEca = ([urine calcium] × [serum creatinine])/([serum calcium] × [urine creatinine]).Tubular fractional reabsorption of phosphorus (TRP) was calculated by the formula: TRP = 1-([urine phosphate] × [serum creatinine])/([serum phosphate] × [urine creatinine]); and percent TRP was calculated by multiplying TRP by 100. The tubular maximum reabsorption of phosphorus per glomerular filtration rate (TmP/GFR [mg/dL]) can be calculated by the following equation [22]: If TRP is ≤ 0.86 (86%), then TmP/GFR = TRP × (serum phosphate). If TRP is > 0.86 (86%), then TmP/GFR = (0.3 × TRP)/(1–0.8 × TRP)x(serum phosphate) [23]. Urine phosphate/creatinine ratio, percent TRP, and TmP/GFR were found to be normally distributed by Kolmogorov-Smirnov test. On the other hand, eGFR, UACR, urine calcium creatinine ratio, and FEca were not normally distributed, and they were log transformed, termed log(eGFR), log(UACR), log(urine Ca/cr), and log(FEca), respectively.

### Statistical analysis

Baseline characteristics were assessed with standard descriptive statistics. Data were expressed as either mean ± standard deviation or median and interquartile range. For comparisons of differences between groups, analysis of variance (ANOVA) was used for variables with normal distribution, and Kruskal-Wallis test was used when data were not normally distributed. To assess the correlation between two variables, a Pearson’s correlation test was used to assess the correlation between two normally distributed variables; for variables that were not normally distributed, a Spearman rank correlation test was used. For multivariate analysis, multivariate linear regression and multivariate logistic regression analyses were used. Data analysis was performed by SPSS statistics version 22.0 (IBM, Armonk, NY). A value of P < 0.05 was taken to be statistically significant.

## Results

### Patient characteristics

Among the 185 patients enrolled in the study, 135 were male (73%). More than half of the study subjects were taking ACE inhibitors and/or AT1 receptor blockers (Table 1). Of the 185 patients, 44 (24%), 124 (67%), and 17 (9%) had an eGFR (mL/min/1.73 m^2^) of ≥60, between 30 and 60, and < 30, respectively. In addition, 135 (73%), 40 (22%), and 10 (5%) patients had a UACR (mg/g•cr) of < 30 (normoalbuminic range), between 30 and 300 (microalbuminuric range), and ≥ 300 (macroalbuminuric range), respectively. Serum FGF23 levels between patients who were and were not taking ACE inhibitor and/or angiotensin II receptor blocker did not differ significantly (data not shown). By contrast, α-Klotho levels in patients who were taking ACE inhibitor and/or angiotensin II receptor blocker (median 317, interquartile range 216–427 pg/mL, n = 96) were significantly lower than those in patients who were not taking either drug (median 397, interquartile range 221–520 pg/mL, n = 89) (P = 0.045, by Mann–Whitney analysis).

**Table 1:** **Demographic characteristics of the study patients**

Variables				Variables		
Clinical characteristics				Laboratory measurements	Median	(Interquartile range)
Age	68.9	±	11.3	Complete blood cell count		
Sex (women/men)	50	/	135	White blood cell count. ×10^3^/mL	5.9	(4.9 - 7.0)
Body mass index, kg/m^2^	23.3	±	3.1	Hemoglobin, g/dL	13.5	(12.4 - 14.7)
Systolic blood pressure, mmHg	128.2	±	19.5	Platelet count. ×10^4^/mL	20.1	(17.2 - 25.3)
Cardiovascular disease				Blood chemistry		
Ischemic heart disease, n (%)	124		(67.0)	Total protein, g/dL	6.9	(6.6 - 7.4)
Arrhythmia, n (%)	43		(23.2)	Albumin, g/dL	4.0	(3.8 - 4.2)
Cardiomyopathy, n (%)	22		(11.9)	Alanine aminotransferase, IU/L	19	(14–27)
NYHA class III/IV, n (%)	16		(8.6)	Blood urea nitrogen, mg/dL	17	(14–21)
Aortic aneurysm, n (%)	10		(5.4)	Serum creatinine, mg/dL	0.88	(0.74 - 1.08)
Peripheral artery disease, n (%)	19		(10.3)	eGFR. mL/min/1.73 m^2^		49.5 ± 15.0
Valvular heart disease, n (%)	7		(3.8)	C-reactive protein, mg/dL	0.10	(0.04 - 0.45)
Smoking status				B-type natriuretic peptide, pg/mL	49.4	(22.3 - 123.4)
Never, n (%)	69		(37.3)	Corrected calcium, mg/dL	9.1	(8.8 - 9.4)
Former, n (%)	89		(48.1)	Inorganic phosphate, mg/dL		3.3 ± 0.5
Current, n (%)	27		(14.6)	FGF23, pg/mL	46.9	(33.7 - 67.8)
				α-Klotho, pg/mL	343	(218–474)
Medication						
ACE inhibitors/ARB, n (%)	96		(51.9)	Urine chemistry		
Beta blockers, n (%)	72		(38.9)	Albumin, g/dL	8.7	(4.5 - 34.0)
Calcium channel blockers, n (%)	84		(45.4)	Creatinine, mg/dL	74.8	(42.9 - 126.4)
Aldosterone antagonist, n (%)	8		(4.3)	Albumin/creatinine ratio, mg/g	12.9	(6.6 - 34.4)
Sulfonylurea, n (%)	16		(8.6)	Calcium/creatinine ratio, mg/g	0.07	(0.04 - 0.12)
DPP4 inhibitors, n (%)	28		(15.1)	Phosphate/creatinine ratio, mg/g		0.49 ± 0.21
Insulin, n (%)	15		(8.1)	Fractional excretion of calcium, %	0.73	(0.42 - 1.12)
Loop, n (%)	32		(17.3)	TRP, %		86.1 ± 6.7
Thiazide, n (%)	15		(8.1)	TmP/GFR, mg/dL	2.9	(2.5 - 3.5)
Statin, n (%)	96		(51.9)			

When patients were categorized according to their eGFR levels (Table 2), serum levels of FGF23 and α-Klotho, and parameters related to urine excretion of calcium or phosphate, except urine phosphate creatinine ratio, were significantly different across the groups. For example, α-Klotho, percent TRP and FEca showed a graded decrease, and FGF23 showed a graded increase with the progression of CKD stages. Serum concentrations of FGF23 and α-Klotho in patients with grade 4, but not those in patients with grade 3 CKD, differed significantly from those in patients without CKD (Dunnett’s post-hoc analysis).

**Table 2:** **Laboratory data stratified by eGFR values**

	eGFR ≥60 mL/min/m ^2^		eGFR 30–60 mL/min/m ^2^		eGFR <30 mL/min/m ^2^		
Variables	(n = 44)		(n = 124)		(n = 17)		P value
eGFR, mL/min/1.73 m^2^		69.2 ± 8.7		45.9 ± 8.4		25.6 ± 8.4	<0.001
Urine albumin creatinine ratio	10	(7–55)	13	(6–27)	75	(10–196)	0.036
Serum cCa, mg/dL	9.1	(8.8 - 9.5)	9.1	(8.7 - 9.3)	9.1	(8.7 - 9.3)	0.508
Serum inorganic phosphate, mg/dL	3.40	(3.10 - 3.70)	3.30	(2.90 - 3.70)	3.30	(3.00 - 3.65)	0.546
Urine calcium creatinine ratio, mg/g	0.11	(0.07 - 0.17)	0.07	(0.04 - 0.11)	0.03	(0.01 - 0.05)	<0.001
Fractional excretion of calcium, %	0.81	(0.48 - 1.30)	0.72	(0.42 - 1.05)	0.46	(0.17 - 0.89)	0.041
Urine phosphate creatinine ratio, mg/g		0.50 ± 0.23		0.49 ± 0.20		0.39 ± 0.15	0.149
TRP, %		90.1 ± 4.9		85.5 ± 6.2		80.2 ± 8.6	<0.001
TmP/GFR, mg/dL	3.3	(2.8 - 4.2)	2.8	(2.5 - 3.4)	2.6	(2.2 - 3.2)	<0.001
FGF23, pg/mL	41.2	(29.4 - 62.3)	47.0	(35.3 - 67.9)	66.4	(45.6 - 111.3)	0.009
α-Klotho, mg/dL	425	(254–539)	351	(224–473)	211	(163–286)	0.001

Serum levels of FGF23 or α-Klotho, and the urine parameters described above, except TRP, did not differ according to the albuminuric status (Table 3).

**Table 3:** **Laboratory data stratified by UACR values**

	UACR <30 mg/g cr		UACR 30–300 mg/g cr		UACR ≥300 mg/g cr		
Variables	(n = 135)		(n = 40)		(n = 10)		P value
eGFR, mL/min/1.73 m^2^		51 ± 14		48 ± 17		39 ± 17	0.039
Urine albumin creatinine ratio	9	(5–15)	76	(51–154)	1241	(719–2045)	<0.001
Serum cCa, mg/dL	9.1	(8.8 - 9.4)	9.3	(8.8 - 9.5)	9.3	(8.8 - 9.5)	0.530
Serum inorganic phosphate, mg/dL	3.30	(2.90 - 3.70)	3.30	(2.90 - 3.50)	3.65	(3.30 - 3.98)	0.042
Urine calcium creatinine ratio	0.07	(0.04 - 0.12)	0.07	(0.03 - 0.15)	0.07	(0.02 - 0.10)	0.789
Fractional excretion of calcium, %	0.73	(0.43 - 1.06)	0.71	(0.36 - 1.50)	0.74	(0.38 - 1.33)	0.984
Urine phosphate creatinine ratio		0.47 ± 0.19		0.52 ± 0.23		0.59 ± 0.25	0.088
TRP, %		87.2 ± 5.7		84.0 ± 8.0		80.6 ± 9.6	0.001
TmP/GFR, mg/dL	3.0	(2.6 - 3.5)	2.7	(2.2 - 3.4)	2.9	(2.6 - 3.5)	0.186
FGF23, pg/mL	46.8	(34.3 - 67.0)	44.1	(29.9 - 68.5)	58.3	(47.0 - 86.3)	0.330
α-Klotho, mg/dL	345	(223–468)	330	(200–474)	358	(218–589)	0.736

### Correlation between serum FGF23/α-Klotho and eGFR/UACR and other calcium phosphate metabolism-related factors

**Figure 1:**
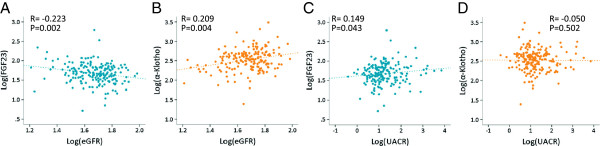
**Scatter plots and correlation coefficients between FGF23 or α-Klotho, and estimated glomerular filtration rate (eGFR) or urine albumin/creatinine ratio (UACR). A**. Correlation between log(eGFR) and log(FGF23). **B**. Correlation between log(eGFR) and log(α-Klotho). **C**. Correlation between log(UACR) and log(FGF23). **D**. Correlation between log(UACR) and log(α-Klotho). Spearman’s correlation analysis was performed.

Log(eGFR) was significantly associated with log(UACR) with a correlation coefficient of −0.190 (P = 0.009). FGF23 was significantly correlated negatively with log(eGFR) and positively with log(UACR) (Figure 1). As we reported before, α-Klotho was not significantly correlated with serum levels of either calcium or phosphate [12]; however,FGF23 showed a positive correlation with serum calcium, which differed from our previous observation [12]. Among the urine parameters, FGF23 was significantly correlated with parameters related to calcium excretion, including log(urine Ca/cr) or log(FEca), but not with parameters of phosphate excretion, namely, urine phosphate creatinine ratio, percent TRP, or TmP/GFR (Table 4). When patients were divided according to their CKD status, a significant association between FGF23 and calcium and between FGF23 and phosphate was observed exclusively among those with CKD (i.e., GFR < 60 mL/min/1.73 m^2^). Among the group of patients with CKD, α-Klotho was significantly correlated with parameters related to calcium excretion, but not with those related to phosphate excretion.

**Table 4:** **Correlation between FGF23/α-Klotho and serum and urine calcium/phosphate-related parameters**

	Total study population				eGFR ≥60 mL/min/m ^2^				eGFR <60 mL/min/m ^2^			
	Log (FGF23)		Log (α-Klotho)		Log (FGF23)		Log (α-Klotho)		Log (FGF23)		Log (α-Klotho)	
Log (FGF23)	-		−0.14	0.064	-		−0.23	0.138	-		−0.10	0.256
Log (α-Klotho)	−0.14	0.064	-		−0.23	0.138	-		−0.10	0.256	-	
Log (cCa)	0.22	0.003	0.06-	0.444	0.13	0.395	0.09	0.553	0.26	0.002	0.05	0.559
Inorganic phosphate	0.23	0.002	0.05	0.509	0.11	0.469	−0.25	0.104	0.27	0.001	−0.01	0.934
Log (urine Ca/cr)	−0.15	0.039	0.22	0.003	−0.23	0.134	−0.05	0.735	−0.09	0.311	0.26	0.002
Log (FEca)	−0.15	0.045	0.15	0.037	−0.25	0.106	−0.04	0.786	−0.10	0.247	0.19	0.021
Urine phosphate creatinine ratio	0.07	0.348	0.03	0.728	−0.09	0.544	−0.13	0.411	0.12	0.153	0.08	0.363
Percent TRP	−0.14	0.064	0.11	0.124	0.15	0.331	0.03	0.858	−0.14	0.099	0.08	0.331
Log (TmP/GFR)	0.07	0.355	0.04	0.634	0.17	0.276	−0.13	0.385	0.10	0.247	0.04	0.638

For the correlation between log(FGF23) and log(α-Klotho) and between log(FGF23) or log(α-Klotho) and inorganic phosphate, urine phosphate creatinine ratio, percent TRP, and log(TmP/GFR), Pearson’s correlation test was used. Spearman’s correlation test was used otherwise.

### Linear regression analysis

Next, we performed univariate and multivariate analyses for patients using log(FGF23) or log(α-Klotho) as an independent variable (Table 5). eGFR was associated with both log(FGF23) and log(α-Klotho); and log(cCa) and inorganic phosphate were associated significantly with log(FGF23), but not with log(α-Klotho), consistent with the correlation analysis. Multivariate stepwise regression analysis showed that age, eGFR, log(cCa), and inorganic phosphate were selected as significant predictor for log(FGF23), and only eGFR was selected as a predictor for log(α-Klotho).

**Table 5:** **Linear regression analysis**

	Univariate model			Stepwise multivariate model		
Predictors	Std β	(95% confidence interval)	P value	Std β	(95% confidence interval)	P value
dependent variable: Log(FGF23)						
Sex (male = 1)	−0.02	(−0.17 - 0.12)	0.741			
Age	0.21	(0.07 - 0.35)	0.004	0.16	(0.01 - 0.30)	0.034
eGFR	−0.21	(−0.35 - -0.07)	0.004	−0.16	(−0.34 - -0.02)	0.027
Log(UACR)	0.13	(−0.01 - 0.28)	0.077			
Log(cCa)	0.24	(0.10 - 0.38)	0.001	0.21	(0.07 - 0.35)	0.003
Inorganic phosphate	0.23	(0.09 - 0.37)	0.002	0.19	(0.05 - 0.33)	0.007
Dependent variable: log(α-Klotho)						
Sex (male = 1)	0.00	(−0.14 - 0.15)	0.955			
Age	−0.14	(−0.28 - 0.01)	0.064			
eGFR	0.24	(0.10 - 0.39)	0.001	0.24	(0.10 - 0.39)	0.001
Log(UACR)	−0.01	(−0.15- 0.14)	0.931			
Log(cCa)	−0.03	(−0.18 - 0.12)	0.680			
Inorganic phosphate	−0.05	(−0.19 - 0.10)	0.509			

We also performed a linear regression analysis after dividing the study population according to their CKD status. None of the parameters tested was selected as a significant predictor for either log(FGF23) or log(α-Klotho) among patients in the no CKD group (Table 6). By univariate analysis, log(UACR) was found to be associated with log(FGF23) among patients with CKD; however, the association did not remain significant in a stepwise multivariate model.

**Table 6:** **Linear regression analysis among no-CKD and CKD groups**

	eGFR ≥60 mL/min/m ^2^			eGFR <60 mL/min/m ^2^					
	Univariate model			Univariate model			Stepwise multivariate model		
Predictors	Std β	(95% confidence interval)	P value	Std β	(95% confidence interval)	P value	Std β	(95% confidence interval)	P value
Dependent varialble: log(FGF23)									
Sex (male = 1)	0.05	(−0.26 - 0.36)	0.743	−0.10	(−0.27 - 0.06)	0.227			
Age	0.08	(−0.23 - 0.39)	0.589	0.23	(0.07-0.39)	0.006	0.24	(0.08 - 0.39)	0.003
eGFR	−0.02	(−0.34 - 0.29)	0.874	−0.19	(−0.35 - -0.02)	0.025			
Log(UACR)	−0.05	(−0.36 - 0.26)	0.742	0.18	(0.01 - 0.34)	0.037			
Log(cCa)	0.22	(−0.08 - 0.52)	0.150	0.24	(0.08 - 0.40)	0.004	0.21	(0.06 - 0.37)	0.008
Inorganic phosphate	0.11	(−0.20 - 0.42)	0.469	0.27	(0.10 - 0.43)	0.001	0.21	(0.05 - 0.37)	0.011
Dependent variable: log(α-Klotho)									
Sex (male = 1)	0.11	(−0.20 - 0.42)	0.474	0.02	(−0.14 - 0.19)	0.794			
Age	−0.17	(−0.48 - 0.14)	0.269	−0.09	(−0.15 - 0.19)	0.794			
eGFR	0.09	(−0.22 - 0.40)	0.564	0.24	(0.08 - 0.40)	0.004	0.24	(0.08 - 0.40)	0.004
Log(UACR)	−0.09	(−0.40 - 0.22)	0.551	0.02	(−0.15 - 0.18)	0.844			
Log(cCa)	0.00	(−0.31 - 0.31)	0.995	−0.03	(−0.20 - 0.13)	0.685			
Inorganic phosphate	−0.19	(0.52 - 0.13)	0.237	−0.01	(−0.17 - 0.16)	0.934			

## Discussion

It was found that serum FGF23 was correlated with both eGFR and UACR in a univariate model (Figure 1). When the study patients were categorized by their eGFR values, FGF23 showed a graded increase and α-Klotho showed a graded decrease according to the progression of CKD stages (Table 2). On the other hand, such an association was not apparent when the patients were divided according to the albuminuric status (Table 3). In the multivariate regression model, eGFR was selected as, respectively, a significant negative and positive predictor for serum FGF23 and α-Klotho; in contrast, UACR was not selected as a predictor (Table 5).

It is increasingly recognized that both a low glomerular filtration rate and increased urinary protein excretion are associated with an adverse cardiac or cardiovascular outcome [24, 25]. Both low eGFR [11, 18] and proteinuria [26] have been shown to be associated with cardiac remodeling. On the other hand, as compared with the relationship between eGFR and FGF23/α-Klotho, information seems to be limited regarding the relationship association between proteinuria (or albuminuria) and FGF23/α-Klotho. Lundberg et al. demonstrated that, among patients with IgA nephropathy, FGF23 was significantly correlated with both albuminuria and eGFR, although they did not explore this relationship in a multivariate model [27]. When we analyzed the patients who did and did not have eGFR values in the CKD range (i.e., < 60 mL/min/1.73 m^2^), FGF23 levels were found to be related to eGFR in those with CKD, but not in those without (Table 5). These findings were consistent with the notion that factors other than eGFR, proteinuria, or inorganic phosphate may have a role in modulating FGF23 levels [15]. In our previous study, we showed that, even among patients without CKD, higher serum FGF23 tended to be associated with lower systolic function [12]; therefore, such factors, if present at all, should be pursued among cardiology patients in future studies.

Klotho is expressed predominantly in the kidney and, to the less extent, in the brain. As described above, previous studies have shown that, in parallel with the reduction of glomerular filtration rate, renal expression of klotho is decreased [5, 6], which may explain the lower circulating α-Klotho level among in patients with low eGFR [4]. These findings are consistent with the finding in the current study, that serum α-Klotho was decreased especially in the patients in the higher CKD grades. On the other hand, α-Klotho was not associated with the extent of proteinuria, regardless of the presence or absence of a decline in renal function in the current study.

Several experimental [28, 29] and human studies [30] suggested the possible relationship between the extent of proteinuria and renal klotho expression. The observation that drug intervention that can potently decrease proteinuria increases renal Klotho expression or circulating α-Klotho levels in aging-related renal injury [31] or diabetic nephropathy [32] may further suggest the possible interaction between Klotho expression or secretion is decreased and proteinuria. On the other hand, Kim et al. reported that eGFR, but not proteinuria, was found to be associated with α-Klotho level using a statistical model that include both eGFR and proteinuria [4]. Karalliedde et al. showed that, although angiotensin II receptor blockage increased soluble Klotho, the extent of albuminuria was not associated with soluble Klotho, in diabetic subjects [33]. Similarly, Lim et al. reported that reduction of proteinuria by angiotensin II receptor blocker, but not by angiotensin converting enzyme (ACE) inhibitor resulted in an increase in the circulating α-Klotho in diabetic patients. These finding may collectively suggest that extent of proteinuria by itself is not be a determinant of circulating α-Klotho, although how angiotensin II receptor blocker cause an increase in α-klotho expression and secretion is unknown [32]. Our data also indicate that serum α-Klotho is related to renal function, but not to the extent of proteinuria, at least among cardiology patients.

We also examined the relationship between FGF23/α-Klotho and urine excretion of calcium and phosphate (Table 4). It was rather unexpected that FGF23 levels were not significantly correlated with TmP/GFR. FGF23 regulates urinary phosphate excretion, as well as production of vitamin D and parathyroid hormone. FGF23 was shown to be positively associated with TmP/GFR in healthy subjects [34], and, in addition, phosphaturic activities of FGF23 have also been convincingly demonstrated in the genetically engineered animal models [35, 36] and in a patient with high FGF23 levels due to tumor-induced osteomalacia [37].

On the other hand, Komo et al. reported in an analysis of data from patients referred to their hospital for osteoporosis treatment, that serum FGF23 was not correlated with inorganic phosphate, corrected calcium, or TmP/GFR [38]. They speculated that association between FGF23 and TmP/GFR may be weaker in the absence of severe renal dysfunction. Consistent with their notion findings, association between FGF23 and TmP/GFR was significant, albeit borderline, among patients with CKD in the current study population (Table 4). Of note, it is known that resistance to the elevated FGF23 may occur in some diseased conditions, such as autosomal dominant polycystic kidney disease [39, 40]. Although the precise mechanism of this resistance remains unclear, decreased renal Klotho expression is presumed to play a role. Whether FGF23 resistance is present among patients with certain cardiac or vascular disorder should be investigated in future studies.

The current study has some limitations. First, ACE inhibitors and/or angiotensin II receptor blockers were prescribed for a substantial proportion of the study subjects, and patients who were taking these medications had lower serum α-Klotho than those who were not. Because this was not a randomized interventional study, however, we are not able to draw conclusions about whether or not these medications increase or decrease serum α-Klotho. Second, we excluded patients who were undergoing chronic hemodialysis; therefore, the relationship between FGF23/α-Klotho and renal function may be underestimated [16].

## Conclusions

Among cardiology patients, eGFR, but not the extent of proteinuria, was independently associated with serum levels of FGF23 and α-Klotho. In addition to clarifying the prognostic importance of these molecules for the prediction of outcome [41, 42], the investigation of factors that are and are not related to FGF23/α-Klotho should be continued in order to discover how to modulate the FGF23/α-Klotho axis for the therapeutic purpose.
